# Global child health in Germany - Time for action

**DOI:** 10.1080/16549716.2020.1829401

**Published:** 2020-10-09

**Authors:** Ralf Weigel, Carsten Krüger

**Affiliations:** a Friede Springer Endowed Professorship for Global Child Health, Faculty of Health, Department of Medicine, Witten/Herdecke University, Witten, Germany; b Faculty of Health, Department of Medicine, Division of Paediatrics, Witten/Herdecke University, Witten, Germany; c Department of Applied Health Sciences, University of Applied Sciences, Hsg Bochum, Germany

**Keywords:** Adolescent, child, child health, global health, goals, sustainable development, Germany

## Abstract

Child health is central to the SDG agenda. Universities in the UK and other European countries provide leadership in research and education for global child health to inform related policy and practice, but the German contribution is inadequate. German paediatricians and other child health professionals could make more substantial contributions to the debate at home and internationally, but lack opportunities for scholarship and research. We argue, that there is a momentum to advance global child health in academia and call on German universities to realise this potential.

‘Viruses don’t need visas, pathogens don’t need passports’ – the World Health Organization (WHO) Director-General’s urgent message to the participants of the World Health Summit in Berlin in 2017 is more relevant today than ever [1]. The impact of the SARS-CoV-2 pandemic on children is a powerful reminder in this regard [2] and other threats are looming [3,4]. Germany, like other high-income countries, is a beneficiary of globalisation. However, benefits come with responsibilities: as a signatory of the Sustainable Development Goals (SDG) 2016–2030, Germany committed to advance health globally [5]. Child health and well-being are central to the SDG agenda illustrating our responsibility for future generations [6,7]. Unfortunately, global child health in Germany is somewhat neglected in research and education. We need a major effort to improve the situation.

## Barriers to global child health

In Germany, global child health institutions and the scientific debate are still in their infancy compared to other European countries. In the UK (UK), the Centres at the University College London, the London School of Hygiene and Tropical Medicine, the Liverpool School of Tropical Medicine, and other universities have active research groups in global child health as an integral part of maternal, newborn, child and adolescent health. The Royal College of Paediatrics and Child Health annual meetings regularly devote entire days to global child health research and training. Global child health topics regularly feature in the College’s scientific journal. Universities in Italy, the Netherlands, Norway and Sweden have institutes dedicated to international maternal and child health. At the universities in Utrecht, London and Liverpool, under- and postgraduates can attend various courses on global child health. In Sweden, the Institute for Global Health Transformation initiated a multidisciplinary forum hosted by the Royal Swedish Academy of Sciences, which resulted in a roadmap on global child health with five priority areas in the context of the SDGs [8].

Although Germany has successful research groups in maternal and child public health that collaborate internationally, for example at the universities in Hamburg, Heidelberg and Munich, there is no such overarching forum to share ideas, to develop strategies and to provide direction. It is the private Witten/Herdecke University that has the only professorship for global child health, funded by the Friede Springer Foundation [9]. The German Society for Tropical Paediatrics and International Child Health (GTP) is a professional society established almost 40 years ago with about 400 current members which brings together paediatricians with different backgrounds at its annual meetings and offers a range of trainings, but its mandate for research is limited [10]. The academic global child health landscape in Germany is fragmented, without a dedicated chair at a state-funded university and with little collaboration between different actors.

However, there are also deeper and more systemic reasons why German global health research and education as a whole are underdeveloped [11,12]. For example, the abuse of public health by the Nazi regime for their racial hygiene policies and atrocities descredited the field and left a stain that still affects perceptions today [13,14]. Currently, public health research is concentrated at several federal institutions, such as the Robert Koch Institute (RKI) and the Federal Centre for Health Education (BZgA). But, compared with universities, their scholarly role is limited. At the local level, public health interventions are implemented by public health offices that have no formal academic role [15]. Furthermore, global health policy programmes in Germany are distributed over six ministries and international health programmes are funded to a large degree not by the Ministry of Health but the Federal Ministry for Economic Cooperation and Development. Its main implementers, the Society for International Cooperation (GIZ) and the Kreditanstalt für Wiederaufbau (KfW), a promotional bank owned by the state, have little focus on academic research and education. Thus, the historical heritage, and the policy and funding structure appear to be barriers that may have contributed to the slow development of an academic base in global health in general [16] and global child health specifically.

## Opportunities for improving global child health in Germany

Within this historical and structural context and with weakly organised public or global health institutions, it is not surprising that German paediatricians are hard to find in scientific landmark publications, guidelines and reports of global relevance. In the 108-page Global Strategy 2016–2030 for the Health of Women, Children and Adolescents [17], a ground-breaking document for the global health of mothers and children, no German name is found in the recognition and author lists, similar to the 16 review articles in the BMJ Special Issue 2015, which provides the scientific background for the strategy [18]. Of the 471 organisations that contributed to the development process of the strategy, only five came from Germany [19]. The same applies to the WHO publications ‘Standards to improve the quality of care for mothers and newborn babies in health care institutions’ from 2016 [20] and ‘Standards to improve the quality of care for children and adolescents in health care institutions’ from 2018 [21]. Among the authors from more than 100 institutions, only three and seven, respectively, are from Germany, and only in one case from a paediatric professional society. Similarly, of the institutions involved in ‘The 2019 report of the Lancet Countdown on health and climate change: ensuring that the health of a child born today is not defined by a changing climate’, 10 come from the US, 11 from the UK, five from other European countries, including one from Germany, and five from other, non-European countries [4]. Although this lack of representation is not necessarily a sign of a lack of participation in the international scientific debate, the few opportunities German researchers have to engage in global child health research and education at universities suggest that this is, in fact, the case. Without academic leadership, a lively exchange of ideas, a research agenda and funding, it is hard to participate and to be heard. Without global child health institutes, students and young researchers have few opportunities and academic career prospects, preventing them from engaging in research and applying for funding. Our research and educational institutions need to provide a better environment for child health professionals that they can move the global scientific and policy debate forward and contribute more substantially to the global research agenda.

Many opportunities exist for paediatricians and other health workers caring for children to engage with the realities of global child health in research and education. For example, in 2015/2016, some 350,000 children and their families came to Germany to seek refuge, many of them vulnerable with multiple risks and in urgent need of health care [22,23]. Their physical and mental health needs and strategies to meet them are important to share [24,25]. What are the enablers and barriers to their integration in the health care and education system, viewed from a child rights perspective [26]? Germany’s development cooperation focus on health systems strenghtening offers further opportunities. The initiative ‘Hospital Partnerships – Partners Strengthen Health’ financed by the Federal Ministry for Economic Cooperation and Development and the Else Kröner-Fresenius Foundation, supports 181 projects with institutions from 51 low- and middle-income countries, several of them focusing on mother and child health [27]. The German Academic Exchange Service (DAAD) has helped to establish 28 cooperations between universities in Germany and low- and middle- income countries with its ‘Partnership for Health Care in Developing Countries’ programme [28], some addressing maternal and child health. Rigorous evaluation of the short and long term effects of interventions implemented within these partnerships, for example on human resources or on child health outcomes, would also make a substantial contribution to the field.

It is time for German universities to use this potential to strengthen research and education in global child health – there is momentum to realise this. The SARS-CoV-2 pandemic has fuelled a debate of how social determinants, such as access to education, affect health, well-being and development of children in Germany and elsewhere [29,30]. Children are leading in advocacy for their own for their right to health in the context of climate change, holding world leaders accountable in the Fridays-for-Future movement. The experiences of families while educating their children at home during lock-downs due to the pandemic as well as the voices of children concerned about climate change are making headlines in the media [31,32]. This may represent an opportunity to leverage global child health concepts, such as social and environmental determinants of health and child rights, higher on the policy and research agenda.

As Germany is updating its global health strategy, receiving valuable advice from various professional organisations [33,34], global child health has to become a core element of this strategy, building on and developing further existing initiatives. A recent discussion paper, published by the Commission for Global Child Health of the German Academy for Child and Adolescent Medicine (DAKJ), listed several recommendations for improving the landscape of global child health research and education [35]. In addition, the German Society of Tropical Paediatrics and International Child Health and the named DAKJ commission will continue to lobby for the inclusion of global child health into the planned German Centre for Child Health, funded by the Federal Ministry of Education and Research [36]. And the recently founded Global Health Hub Germany [37] and the German Alliance for Global Health Research [38] are also prime opportunities for building institutional capacity.

To date, the global child health agenda has had limited visibility in Germany. We call on the academic leadership of paediatric professional societies in Germany to provide a forum for the scientific and political aspects of global child health, to provide leadership and to lobby for funding from the government. Paediatric researchers should respond more actively to calls from multilateral agencies like WHO [39,40] and make public their positions on issues such as child rights [41]. Medical faculties need to strenghten their academic base by offering under- and postgraduate education in global child health through institutes and chairs so that students and young researchers see a path for their careers. We must now seize the opportunities unfolding for urgently needed engagement in this important field in research and education. German universities can and should play a much more active part in advancing the health and well-being of children throughout the world.

## Paper context

This call to action addresses child health professionals and stakeholders to engage in research and education for global child health at Germany’s higher education institutions. Universities should realise the momentum and recognise the importance of global child health to enable substantial contributions to the scientific and policy debate at the national and global levels.
